# *Morchella esculenta* mushroom polysaccharide attenuates diabetes and modulates intestinal permeability and gut microbiota in a type 2 diabetic mice model

**DOI:** 10.3389/fnut.2022.984695

**Published:** 2022-10-06

**Authors:** Ata Ur Rehman, Nimra Zafar Siddiqui, Nabeel Ahmed Farooqui, Gulzar Alam, Aneesa Gul, Bashir Ahmad, Muhammad Asim, Asif Iqbal Khan, Yi Xin, Wang Zexu, Hyo Song Ju, Wang Xin, Sun Lei, Liang Wang

**Affiliations:** ^1^Department of Biotechnology, College of Basic Medical Science, Dalian Medical University, Dalian, China; ^2^Advanced Institute for Medical Sciences, Dalian Medical University, Dalian, China; ^3^Department of Biology, University of Haripur, Haripur, Pakistan; ^4^Stem Cell Clinical Research Center, National Joint Engineering Laboratory, Regenerative Medicine Center, The First Affiliated Hospital of Dalian Medical University, Dalian, Liaoning, China

**Keywords:** *Morchella esculenta*, polysaccharide, streptozotocin, type 2 diabetes mellitus, gut microbiota

## Abstract

Type 2 diabetes mellitus (T2DM) is a health issue that causes serious worldwide economic problems. It has previously been reported that natural polysaccharides have been studied with regard to regulating the gut microbiota, which plays an important role in T2DM. Here, we investigate the effects of *Morchella esculenta* polysaccharide (MEP) on a high-fat diet (HFD) and streptozotocin (STZ)-induced T2DM in BALB/c mice. The administration of MEP effectively regulated hyperglycemia and hyperlipidemia and improved insulin sensitivity. We also determined an improvement in gut microbiota composition by 16sRNA pyrosequencing. Treatment with MEP showed an increase in beneficial bacteria, i.e., *Lactobacillus* and *Firmicutes*, while the proportion of the opportunistic bacteria *Actinobacteria, Corynebacterium*, and *Facklamia* decreased. Furthermore, the treatment of T2DM mice with MEP resulted in reduced endotoxemia and insulin resistance-related pro-inflammatory cytokines interleukin 1β (IL-1β), tumor necrosis factor-alpha (TNF-α), and interleukin 6 (IL-6). Moreover, MEP treatment improved intestinal permeability by modulating the expression of the colon tight-junction proteins zonula occludens-1 (ZO-1), occludin, claudin-1, and mucin-2 protein (MUC2). Additionally, MEP administration affects the metagenome of microbial communities in T2DM mice by altering the functional metabolic pathways. All these findings suggested that MEP is a beneficial prebiotic associated with ameliorating the gut microbiota and its metabolites in T2DM.

## Introduction

Type 2 diabetes mellitus (T2DM) is a chronic metabolic syndrome associated with hyperglycemia, impaired insulin sensitivity, low-grade inflammation, beta-cell failure, and impaired metabolism of glucose, proteins, and lipids (1). Disorders of the kidney, gut, liver, blood vessels, heart, and nerves, are frequently the result of uncontrolled hyperglycemia (2). The etiology of T2DM has genetic and environmental factors (e.g., unhealthy lifestyle, obesity, smoking, and lack of exercise) (3). Several oral anti-diabetic drugs alpha-glucosidase inhibitors, thiazolidinediones, and biguanides reduce hyperglycemia, but have adverse side effects such as flatulence, hypoglycemia, and islet cell damage (4).

Recently, polysaccharides derived from natural resources showed the potential to control glucose and lipid metabolism (5). Some have been investigated for their anti-diabetic effects through insulin enhancement and targeting beta-cell dysfunction, alpha-glucosidase, and inhibition of alpha-amylase (6). Polysaccharides from natural resources have been investigated for regulating gut microbiota through fermentation, resulting in the beneficial metabolites such as short-chain fatty acids (SCFAs). Consequently, the perturbation of gut microbiota regulation and their metabolites by the administration of polysaccharides is considered a potential target for treating diabetes (7).

*Morchella esculenta* is an edible mushroom known for its delicious taste and high nutritional value (8). Consumption of the *Morchella* species as a remedy for various diseases, has been part of traditional Chinese medicine in Malaysia and Japan for over 2,000 years (9). *Morchella esculenta* consists of a variety of bioactive ingredients: proteins, polysaccharides, vitamins and dietary fiber (10), and its polysaccharides have been reported for their anti-proliferation potential against human colon cancer (HT-29 cells) (11). *Morchella esculenta* has also been evaluated for its hepatoprotective potential in mice because of its antioxidation and anti-hyperlipidemic properties (12). *Morchella esculenta* exopolysaccharides have shown an excellent tumor-suppressive effect *in vitro* (13) and immunostimulatory activity by activating T-cells and proliferating splenocytes (14). Moreover, its heteropolysaccharides have strong antioxidant activity and protect zebrafish embryos from oxidative stress (15).

The anti-diabetic activities of *M. esculenta* polysaccharide (MEP) through modulation of gut microbiota, reversal of insulin resistance, and improved intestinal permeability are still unexplored. Therefore, the biological role of *M. esculenta* polysaccharides needs to be explored for its improvement in gut microbiota and protective effect on gut permeability for T2DM.

## Materials and methods

### Chemicals and reagents

The mushroom *M. esculenta* fruiting body was bought from Shandong Tai’an Yinsheng Food Co., Ltd., Shandong, China. Streptozotocin (STZ) was purchased from Sigma Chemical Co. (St. Louis, MO). High-fat diet (HFD) (45% fat Kcal%) was purchased from MediScience Ltd. (Yangzhou, China). The stool DNA isolation kit (FORGENE) was provided by Chengdu, China, and the gel purification kit (Agencourt AMPure XP 60 mL Kit) was obtained from Beckman Coulter (Brea, CA, USA). The bicinchoninic acid (BCA) protein assay kits from TransGen Biotech Co., Ltd., Beijing, China. All ELISA (enzyme-linked immunosorbent assay) kits were purchased from Shanghai Longton Biotechnology Co., Ltd., in Shanghai. The primary antibodies [goat anti-rabbit, β-actin, claudin-1, zonula occludens-1 (ZO-1), occludin and mucin-2 (MUC2)] secondary antibodies, and the Radioimmunoprecipitation assay (RIPA) buffer were purchased from Proteintech (Wuhan, China). All other chemicals were of analytical grade and obtained from standard commercial sources.

### Crude polysaccharides *Morchella esculenta* polysaccharide extraction from mushroom *Morchella esculenta*

The crude polysaccharide from *M. esculenta* mushroom fruiting bodies was extracted according to the protocol previously reported (16). Briefly, the dried fruiting bodies were crushed into a fine powder and mixed with distilled water at a ratio of 1:50 g/ml, then boiled at 80°C for 3 h. The mixture was deproteinized with trichloroacetic acid at 1.5% (v/v), and the pH was adjusted to 7.0 by 2 M (NaOH) followed by centrifugation for 10 min at 10,000 × g. The supernatant was collected and concentrated by rotary evaporation at 65°C. The concentration of protein was measured by BCA quantification. The concentrated solution was precipitated by 3 volumes of ethanol at 4°C for 12 h and then freeze-dried under freeze-drying vacuum systems.

### Monosaccharide composition, protein, and sugar content of *Morchella esculenta* polysaccharide crude polysaccharides

The carbohydrate content of MEP was determined through the phenol-H_2_SO_4_ method (17), and to determine monosaccharide composition, high-performance liquid chromatography (HPLC) was used (18). In brief, 50 mg of purified polysaccharide powder was hydrolyzed at 120°C for 6 h with 2 mol/L of trifluoroacetic aqueous solution. After hydrolysis, the excess acid was removed by co-distillation with methanol to yield a dry hydrolysate, which was dissolved in NaOH and methanol solutions and then incubated for 1 h at 70°C. After pH normalization, distilled water was added and the mixture was extracted three times with chloroform and filtered through a 0.22 μm nylon membrane (Westborough, MA, USA).

### Animals and study design

**FIGURE 1:**
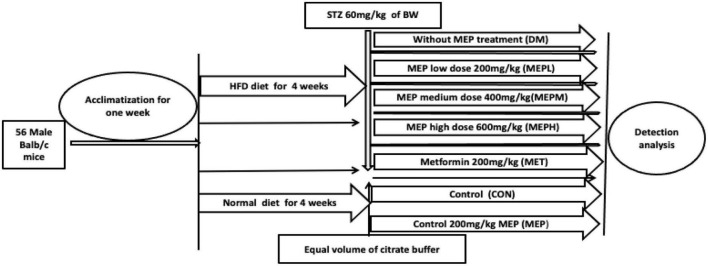
Experimental plan and design. The TD2M mouse model was developed by giving a HFD for 4 weeks, followed by intraperitoneal injections of STZ at 60 mg/kg on an overnight fast every fifth day for 8 weeks. The T2DM mice were divided into five groups (*n* = 8 in each group): no MEP treatment: 200 mg/kg metformin (MET); 200 mg/kg MEP low dose (MEPL); 400 mg/kg MEP medium dose (MEPM); and 600 mg/kg MEP high dose (MEPH). The 16 control mice were divided into two groups (*n* = 8). One was given PBS as control (CON), and the other was given 200 mg/kg MEP.

Fifty-six, four-week-old inbred male BALB/c mice (14 ± 2 g) were obtained from the specific-pathogen-free (SPF) animal care center at Dalian Medical University. The experiment was approved by the Animal Care and Research Ethics Committee, Dalian, China (approval number: ARYX 2019–2021). All mice were acclimatized for 1 week at 22 ± 2°C and 50 ± 10% relative humidity under a 12/12 h light/dark cycle. After acclamation, 16 mice were fed a normal chow diet while the other 40 were fed an HFD (45% fat Kcal%) for 4 weeks. The HFD diet mice were randomly divided into five groups (*n* = 8 in each group) (Figure 1) and injected intraperitoneally with STZ prepared in citrate buffer (pH4.5) at a dose of 60 mg/kg in a fasting condition every fifth day for 4 weeks. The mice on the normal chow diet were injected with an equivalent volume of citrate buffer (19). Fasting blood glucose (FBG) was measured weekly with a glucometer, and mice that had blood glucose levels above 11.1 mmol/L were considered to have T2DM.

After the induction of T2DM, the different groups were made as previously studied (20) and treated as follows: non-MEP treated group (DM); 200 mg/kg metformin (MET); 200 mg/kg MEP low dose (MEPL); 400 mg/kg MEP medium dose (MEPM) and 600 mg/kg MEP high dose (MEPH). Control mice were kept in two groups (*n* = 8 in each). One was given PBS, and the other was given MEP 200 mg/kg (MEP). The study design and schematic presentation are shown in Figure 1. The body weights of all mice were weighed daily to adjust the MEP dosage, while food and water intake were also measured. After 4 weeks of MEP administration, stool samples were collected in sterile eppendorf (EP) tubes and stored immediately at –80°C.

### Determination of fasting blood glucose, oral glucose tolerance test, serum insulin level, and homeostasis model of assessment of insulin resistance

FBG was checked by glucometer weekly throughout the experiment by puncturing the tail vein of overnight fasted mice. An OGTT was performed at the end of the experiment. Briefly, a dosage of 0.2 g/kg of glucose was given by gavage to overnight-fasted mice and blood glucose levels were measured at different intervals: 0, 30, 60, 90, and 120 min by a blood glucometer (Bayer, Leverkusen, Germany) as instructed by the manufacturer. After 4 weeks, the mice were sacrificed by cervical dislocation. Blood was collected through the extirpating eyeball, and serum was obtained by centrifuging at 1,000 × g for 10 min. The fasting serum insulin concentration was determined by a commercially available ELISA kit, according to the manufacturer’s guidelines. Furthermore, the homeostasis model of assessment of insulin resistance (HOMA-IR) was calculated to measure the insulin sensitivity of T2DM mice by using the following formula: HOMA-IR = fasting plasma insulin mIU/L × fasting serum glucose mmol/L/22.5.

### Gut microbiome genomic DNA extraction and 16S rDNA amplicon pyrosequencing analysis

Bacterial genomic DNA samples were isolated using the Power Max (stool/soil) DNA isolation kit (MoBio Laboratories, Carlsbad, CA, USA) and stored at –20°C before further analysis. The isolated genomic DNA was measured using a NanoDropND-1000 (Thermo Fisher Scientific, Waltham, MA, USA), and the quality was evaluated using agarose gel electrophoresis and a spectrophotometer. The 16srRNA gene V4 region was amplified by PCR using the forward primer 515F (5′-GTGCCAGCMGCCGCGGTAA-3′) and the reverse primer 806R (5′-GGACTACHVGGGTWTCTAAT-3′) with the following protocol: Thermal cycling of 30 s of initial denaturation at 98°C, followed by 25 cycles of 15 s of denaturation at 98°C, 15 s of annealing at 58°C, and 15 s of extension at 72°C, with a final extension of 1 min at 72°C.

PCR amplicons were purified with Agencourt AMPure XP beads kit (Beckman Coulter, Indianapolis, IN) and quantified using the Pico Green dsDNA Assay Kit (Invitrogen, Carlsbad, CA, USA). The amplicons were then pooled in a normalized manner and sent for sequencing with a pair-end of 2 × 150 bp by using the IllluminaNovoSeq6000 platform GUHE Info Technology Co., Ltd. (Hangzhou, China). The Quantitative Insights of Microbial Ecology (QIIME, v1.9.0) pipeline was used to analyze the sequence data (21), and valid sequences were identified by removing low-quality reads. An operational taxonomic unit (OTU) was chosen, which included dereplication, clustering, and chimera detection using V search V2.4.4. Subsequently, OTU taxonomy classification was performed with the representative sequence by the Green Gene Database. Moreover, OTU-level alpha, Shannon and Simpson diversities, evenness, and richness index were performed by using QIIME and R packages (v3.2.0), e.g., “vegan.” The beta diversity was performed through UniFrac distance metrics and visualized via principal coordinate analysis (PCoA), non-metric multidimensional scaling (NMDS), and principal component analysis (PCA) (21).

### Metagenomic functional profiling analysis

The analysis of the metagenomic functional profile was assessed using the Phylogenetic Investigation of Communities by Reconstruction of Unobserved States tool (PICRUSt) against the Green Genes database, Kyoto Encyclopedia of Genes and Genomes (KEGG) genes and clusters of orthologous (COG) pathways. The statistical analysis of metagenomic profiles (STAMP) software package version, 2.1.3 was used for functional evaluation. Moreover, to make a figure ligand of ecologically related metabolites and functions in prokaryotic clades (e.g., genera or species), the FAPROTAX database was used (22).

### Determination of pro-inflammatory cytokine concentration in serum

The serum concentration of the pro-inflammatory cytokines interleukin 1β (IL-1β), tumor necrosis factor-alpha (TNF-α), and interleukin 6 (IL-6) were determined by using a mouse ELISA kit (Shanghai Longton Biotechnology Co., Ltd., Shanghai, China), following the manufacturer’s guidelines.

### Western blot analysis

Total protein was extracted from colonic tissue using RIPA lysis buffer containing a protease inhibitor and centrifuged at 12,000 × g for 5 min at 4°C. The protein was quantified by the BCA protein assay kit. Thirty micrograms was fractionated by SDS-PAGE at 8–12% and electroblotted onto polyvinylidene difluoride (PVDF) membranes, which were then blocked for 1.5 h at room temperature in Tris Buffered Saline (TBS) and incubated overnight at 4°C with their respective primary antibodies (1:500–1,000) while β-actin (1:5,000) was loaded as a control. Membranes were washed three times with TBS and incubated with HRP-conjugated secondary antibodies (1:5,000) at room temperature for 1.5 h. The protein bands were exposed to the enhanced ECL chemiluminescent substrate and visualized using an automated imaging system.

### Histopathological and immunohistochemical analysis

Distal colonic tissue (4 μm thick) were fixed in 4% paraformaldehyde at room temperature for 24 h, dehydrated with gradient alcohol, and embedded in paraffin after xylene vitrification. The sections were prepared by microtome (Thermo Fisher Scientific, Waltham, MA, USA), then deparaffinized, rehydrated, and stained with H and E (hematoxylin and eosin) for histological examination. To perform immunohistochemistry (IHC), the deparaffinized and rehydrated section of tissue was incubated for 10 min with 3% H_2_O_2_. In addition, the tissue slide was heated in antigen retrieval buffer (Na^+2^ EDTA, pH 8.0) for antigen retrieval. Then, it was incubated overnight with MUC2-specific primary antibody at 4°C followed by horseradish peroxidase (HRP)-conjugated secondary antibody at room temperature for 1 h. 3,3 diaminobenzidine (DAB) was used as a substrate and hematoxylin was applied as counterstain. Then the slides were mounted and examined under the light microscope at 20 × magnification.

### Statistical analysis

GraphPad Prism (6.01) (La Jolla, CA, USA) was used to analyze all the statistical data. One-way analysis of variance (ANOVA) was performed with Tukey’s multiple comparison test to determine the significance of differences, and a *p*-value of 0.05 was considered statistically significant. LEfSe analysis was performed by Kruskal–Wallis, and Wilcoxon tests. Statistical analysis of the OTUs and phenotype was carried out by a Mann–Whitney test.

## Results

### Characterization and chemical analysis of *Morchella esculenta* polysaccharide

**FIGURE 2:**
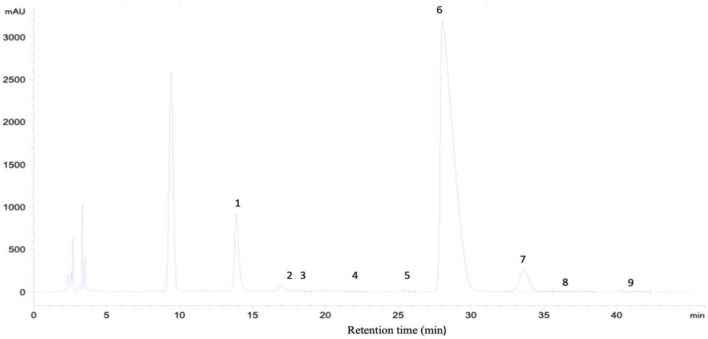
Characterization of MEP and monosaccharide composition analysis by HPLC, chromatogram of standard monosaccharide, 1-mannose, 2-ribose, 3-rhamnose, 4-glucuronic acid, 5-galacturonic acid, 6-glucose, 7-galactose, 8-arabinose, and 9-fucose.

The concentration of polysaccharide (MEP) was found to be 11.96 mg/mL using the phenol-sulfuric acid method and D-glucose as a standard. The yield was 13.5% with total polysaccharide and protein concentrations of 96 and 2.26%, respectively. In comparison to previously analyzed polysaccharides from mushrooms, the yield was 13.2%, with total polysaccharides accounting for 96.66% and protein 2.38% (16). For monosaccharide analysis, the HPLC spectrum indicated that MEP was composed of mannose, ribose, rhamnose, glucuronic acid, galacturonic acid, glucose, galactose, arabinose, and fucose as presented in Figure 2 and Table 1.

**TABLE 1:** Monosaccharide composition of *M. esculenta* crude polysaccharide (MEP).

Components	Concentration mg/kg	Percent
Mannose	2334.69	5.77
Ribose	1924.99	0.263
Rhamnose	86.36	0.018
Glucuronic acid	261.74	0.036
Galacuronic acid	42.91	0.006
Glucose	596,658.04	81.35
Galactose	25,981.32	3.543
Xylose	ND	ND
Arabinose sugar	65,966.00	8.99
Fucose	113.73	0.016

### Effects of *Morchella esculenta* polysaccharide on body weight, glucose tolerance, and insulin resistance

**FIGURE 3:**
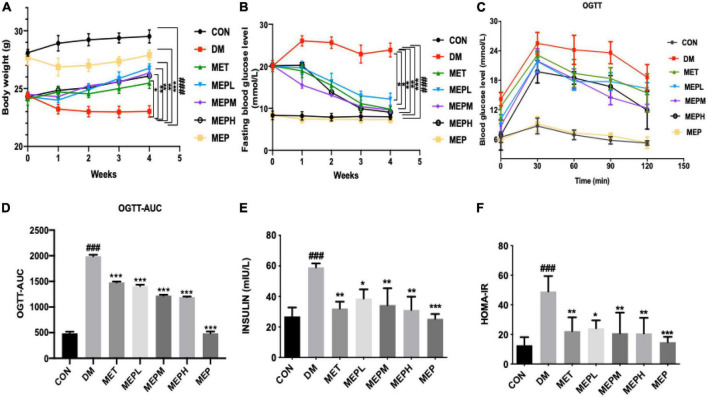
**(A)** The bodyweight of mice was measured daily during MEP treatment **(B)** FBG level, **(C)** OGTT, **(D)** The trapezoidal rule was used to calculate the AUC of OGTT, **(E)** serum insulin level, and **(F)** HOMA-IR was measured after T2DM treatment with MEP in different groups. # indicates a significant difference compared with the control group. ^###^*p* < 0.001 vs. control. *indicates significant difference compared with DM group. *p < 0.05, **p < 0.01 and ***p < 0.001 vs. DM. Data were presented as mean ± standard error of mean (SEM).

We found that the STZ-induced T2DM mice exhibited gradual emaciation and decreased body weight, while the control mice showed a gradual increase in body weight. The MEP and metformin administration for 4 weeks significantly reduced the loss of body weight compared to the DM group. Interestingly, the body weight of the MEP group was lower than that of the control, indicating that MEP administration might have had an impact on normal body weight control (Figure 3A). T2DM is characterized by impaired glucose tolerance, elevated FBG, and increased insulin levels and resistance. An oral glucose tolerance test (OGTT) was performed after 4 weeks of treatment with MEP. FBG and serum fasting insulin levels were measured in each group. The DM group exhibited the highest FBG (23.1 mmol/L), while the MEPM group had the lowest (13.6 mmol/L). FBG levels in the MET, MEPL, MEPM, and MEPH groups were lower compared to that in the DM group (Figure 3B).

The oral glucose tolerance ability of the T2DM mice was severely impaired in the DM group. The blood glucose level at different time intervals remained higher than that of the control group and in the MEP-treated group. The area under the curve (AUC) of OGTT in the DM group was significantly greater, but the AUCs of the control and MEP-treated groups were significantly lower. The MEP effect on OGTT is shown in Figures 3C,D. Fasting serum insulin levels increased significantly in the DM group, representing insulin resistance. However, fasting serum insulin levels decreased significantly in T2DM mice treated with metformin and low, medium, and high doses of MEP compared to the DM group (Figure 3E). The DM group’s homeostasis model assessment-insulin resistance (HOMA-IR) index was significantly higher than that of the control group. However, the metformin and MEP-treated groups MEPL, MEPM, and MEPH HOMA-IR indices were significantly reduced compared to those of the DM group (Figure 3F).

### *Morchella esculenta* polysaccharide effects on bacterial alpha and beta diversity indices in type 2 diabetes mellitus

**FIGURE 4:**
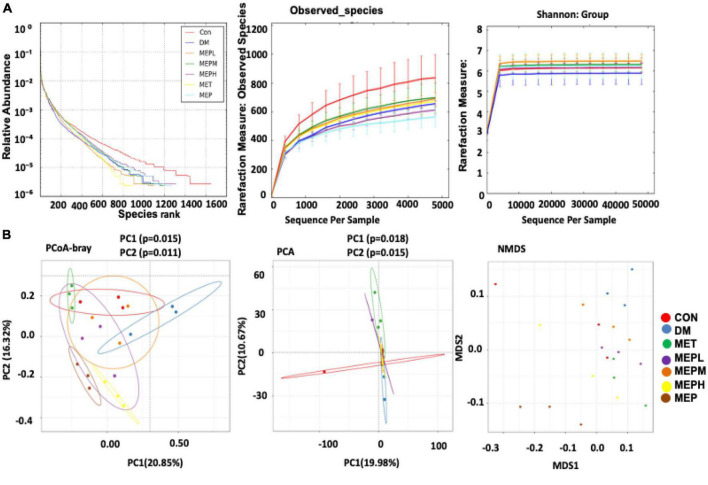
Effect of MEP on bacterial diversity indices (alpha and beta) in T2DM mice. **(A)** The rank abundance curve, observed species, and Shannon show alpha diversity. The rank abundance curve represents bacterial abundance and richness, respectively, while the rarefaction measure of the observed species and Shannon index show species diversity, evenness, and abundance. **(B)** The beta diversity indices were analyzed by the PCoA, PCA, and NMDS plot. Every point represents each sample individually. Points with different colors indicate different treatment groups. The distance between the different points represents the differences and similarities within the bacterial community structure.

An amplicon pyrosequencing platform was employed to explain the overall composition of the gut microbial community in T2DM and control mice after MEP treatment (16S rRNA gene). The alpha and beta diversity of each group was analyzed to evaluate bacterial diversity, abundance, richness, and structural differences. The alpha diversity pattern was analyzed using a rank abundance curve, observed species, and Shannon index to assess the changes. The relative abundance of alpha diversity indices was found to be altered in the DM group compared to the control (Figure 4A). Moreover, the differences among all groups were confirmed by a box plot (Supplementary Figure 1). However, the treatment of MEP altered the alpha diversity in all treated groups of MEP and metformin, as shown in Figure 4A. Other alpha diversity parameters are summarized in Supplementary Table 1. Our results demonstrated that MEP treatment partially improved the altered alpha diversity indices.

To reveal the structural variation of the bacterial community among different groups, the beta diversity pattern was analyzed by using PCA, PCoA, and a NMDS plot (Figure 4B). Our results showed that DM group subjects were clustered separately from the control group. However, the MEP, metformin, and control groups were all relatively closer than the DM group. Significant differences among the groups were observed for PCA in PC1 (*p* = 0.018), PC2 (*p* = 0.015), and for PCoA in PC1 (*p* = 0.015) and PC2 (*p* = 0.011). These findings strongly suggest that the control and MEP-treated groups had many similarities.

### Bacterial taxonomic composition in *Morchella esculenta* polysaccharide-treated type 2 diabetes mellitus mice

**FIGURE 5:**
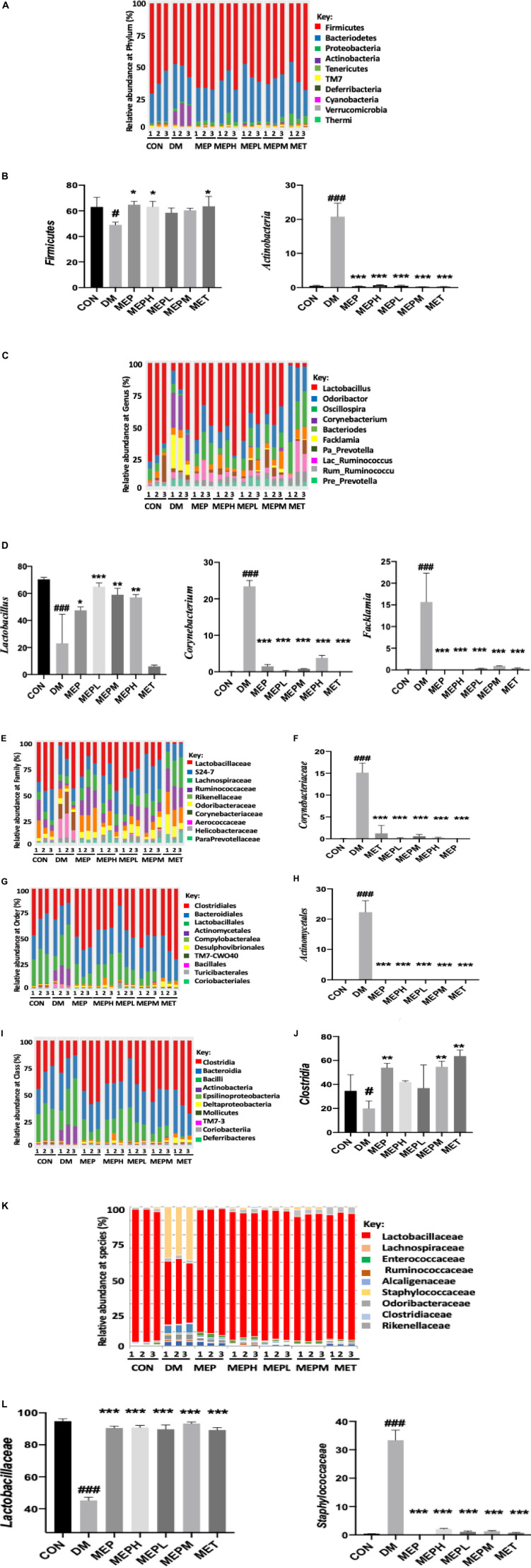
Different taxonomic levels of microbial composition in T2DM mice. **(A)** Relative abundance at the phylum level, **(B)** Abundant flora at the phylum level (%), **(C)** Relative abundance at the genus level, **(D)** Abundant flora at the genus level (%), **(E)** Relative abundance at the family level, **(F)** Abundant flora at the family level (%), **(G)** Relative abundance at the order level, **(H)** Abundant flora at the order level (%), **(I)** Relative abundance at the class level, **(J)** Abundant flora at the class level (%), **(K)** Relative abundance at the species level, and **(L)** Abundant flora at the species level (%). ^#^ indicates a significant difference compared with control (CON) group. ^#^*p* < 0.05, and ^###^*p* < 0.001 vs. Control. * indicates a significant difference compared with the DM group. *p < 0.05, **p < 0.01, and ***p < 0.001 vs. DM.

To reveal whether the MEP administration improved gut microbial composition and structure, OTUs within the range of 592–1289 were analyzed from 21 samples by IllluminaNovoSeq6000. OTU sequencing data are summarized in Supplementary Table 2. At the phylum level, alteration occurred in three major phyla: *Firmicutes, Proteobacteria*, and *Actinobacteria* in the DM group (Figure 5A). *Firmicutes* were reduced (DM, 49.3% vs. control, 64.07%), while *Bacteroidetes* increased (DM, 33.17% vs. control, 31.05%). However, the proportion of *Actinobacteria* showed a dramatic rise (DM, 16.46% vs. control, 0.67%). MEP treatment effectively reversed the altered pattern in the MEP- and MET-treated groups compared to the DM group as presented in Supplementary Table 3. The increased abundance of *Firmicutes* and lower abundance of *Actinobacteria* in the DM group were statistically significant when compared to the control, MEPH, MEP, and MET groups (Figure 5B).

Furthermore, the bacterial composition of T2DM mice showed variation in the family, genus, order, class and species levels of the DM group (Figures 5C–L). At the genus level, *Lactobacillus, Odoribacter, Oscillospira, Corynebacterium, Bacteroides, Facklamia*, and *Prevotella* were found to be abundant flora. Nonetheless, the MEP administration ameliorated the bacterial flora, which had been disturbed to various degrees. *Lactobacillus* decreased drastically (DM, 32.09% vs. control, 69.12%). After MEP and MET treatment, the *Lactobacillus* levels were enhanced: MET (47.41%), MEPL (67.64%), MEPM (64.13%), MEPH (59.12%), and MEP (61.62%) vs. the DM group (32.09%). On the other hand, *Bacteriodes, Corynebacterium*, and *Facklamia* were observed to be higher in the DM group compared to the other groups (Figure 5C and Supplementary Table 4). The DM group showed significantly lower abundance of *Lactobacillus* and higher abundance of *Corynebacterium* and *Facklamia* compared to control and MEP-treated groups (Figure 5D).

Moreover, at the family level, the alteration of the bacterial communities of *Lactobacillaceae, Lachnospiraceae*, and *Enterobacteriaceae* were less abundant and *Corynebacteriaceae* was more abundant in the DM group (Figure 5E and Supplementary Table 5). At the class and order level, *Actinobacteriace* and *Actinomyceletes* increased markedly in the DM group (Figures 5G,I). The statistically significant taxa were observed at the family level *Corynebacteriaceae*, at the class level *Actinomyceletes*, and at the order level *Clostridia* (Figures 5F,H,J). It is noteworthy that after MEP treatment, the bacterial taxonomy was reversed at the species level. As compared to the control, MEP-, and MET-treated groups, the DM groups had an increased abundance of *Staphylococcaceae* and a lower abundance of *Lactobacilliaceae* (Figures 5K,L).

The heat map of microbiome composition with taxonomic-level analysis was clustered following a degree of similarity among all groups (Supplementary Figure 2). Conversely, the relative abundance of perturbed bacterial communities showed amelioration following MEP treatment. In conclusion, our findings revealed that, at the taxonomic level, the bacterial communities of T2DM mice were altered. However, the MET and MEP treatments in all groups improved the composition of the gut microbiota of T2DM mice, and the majority of the flora reverted to normal.

### *Morchella esculenta* polysaccharide effect on the functional profile of gut metabolites in *Morchella esculenta* polysaccharide-treated type 2 diabetes mellitus mice

**FIGURE 6:**
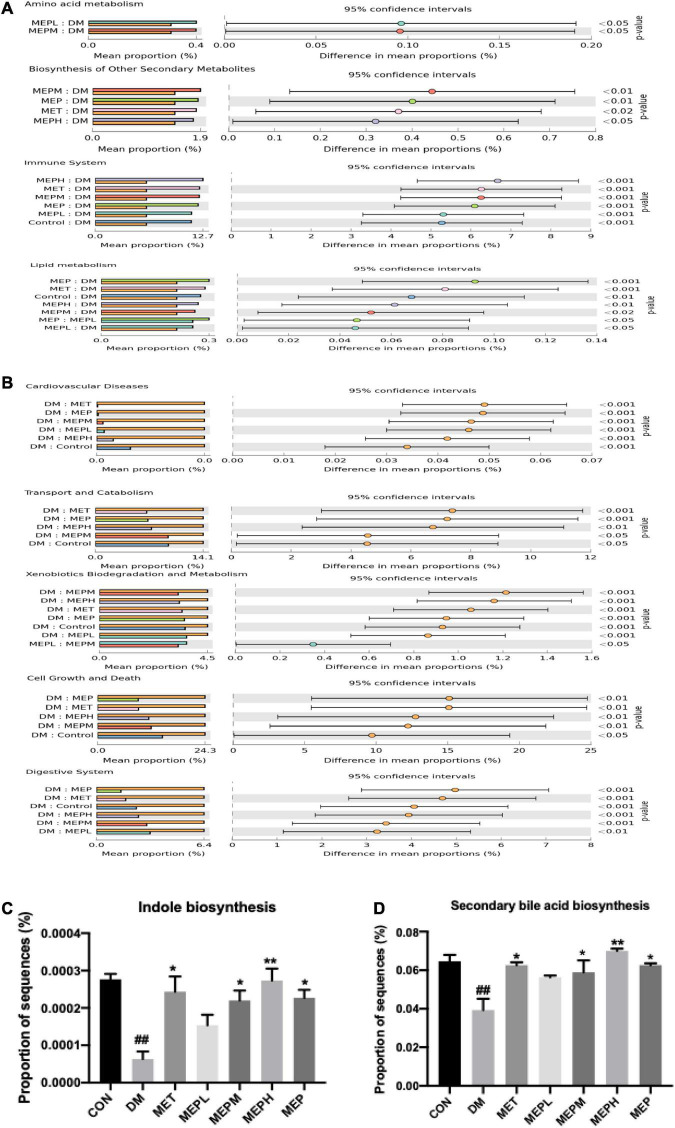
Functional profile analysis of gut metabolites in T2DM mice. **(A,B)** COG (Clusters of Orthologous Groups) shows the metabolic pathways functional predictions and KEGG database level 3 indicated with their confidence interval ratio of differences, while *p*-value shows on the right side among different groups. **(C)** Expression of genes involved in indole biosynthesis and **(D)** Secondary bile acid biosynthesis. ^#^ indicates a significant difference compared with control (CON) group. ^##^*p* < 0.01 vs. Control. * indicates a significant difference compared with the DM group. *p < 0.05, **p < 0.01 vs. DM.

We further determined the effect of the MEP on the metagenome of microbial communities. We found that the DM group showed significant differences in KEGG pathways compared to the control and MEP groups. Our findings demonstrated that amino acid and lipid metabolism, biosynthesis of other secondary metabolites, the immune and endocrine systems, and signaling pathways were found to be downregulated in the DM group, while these pathways were significantly upregulated in the MEP treatment groups (Figure 6A). Furthermore, a metagenomic profile analysis revealed that the DM group had higher levels of gene expression in cardiovascular disease, transport and catabolism, xenobiotic biodegradation and metabolism, cell growth and death, and the digestive system (Figure 6B). Furthermore, the gene expression of indole biosynthesis and secondary bile acid biosynthesis were found to be lower in the DM group, while these genes were found significantly highly expressed in the control, MET-, and MEP-treated groups (Figures 6C,D). These results demonstrated an altered functional metagenome among the DM and control groups.

### Effect of *Morchella esculenta* polysaccharide on pro-Inflammatory cytokine levels in type 2 diabetes mellitus mice

**FIGURE 7:**
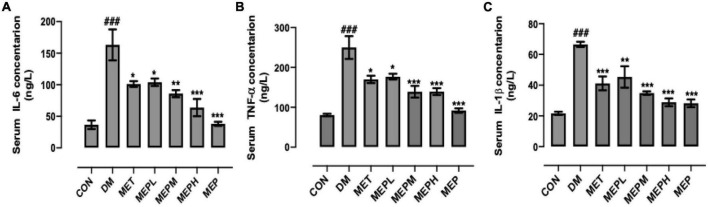
Measurement of pro-inflammatory cytokines **(A)** IL-6, **(B)** TNF-α and **(C)** IL-1β by ELISA in T2DM mice. The data are shown with means ± SEM. ^#^ indicates a significant difference compared with the control (CON) group. ^###^*p* < 0.001 vs. control. * indicates a significant difference compared with the DM group. *p < 0.05, **p < 0.01, and ***p < 0.001 vs. DM.

Serum concentrations of IL-6, IL-1β, and TNF-α were measured in different groups after 4 weeks to determine the immunoregulatory effect of MEP treatment. The DM group showed marked fluctuations compared to the control. Nevertheless, IL-6 decreased significantly in MET or MEPL, MEPM, MEPH, and MEP groups (Figure 7A). Meanwhile, TNF-α also significantly decreased in the MET, MEPL, MEPM, MEPH, and MEP groups (Figure 7B). Furthermore, IL-1β in MET- and MEP-treated groups (MEPL, MEPM, and MEPH) are significantly lower than in the DM group (Figure 7C). These results indicated that MEP treatment has a potential role in reducing T2DM-induced inflammation.

### *Morchella esculenta* polysaccharide reduced type 2 diabetes mellitus-induced metabolic endotoxemia and improved intestinal permeability in type 2 diabetes mellitus mice

**FIGURE 8:**
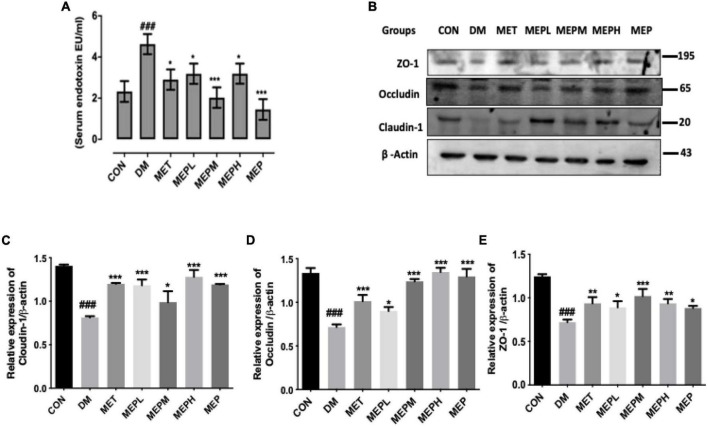
Effect of MEP treatment on serum endotoxin LPS (lipopolysaccharide) levels and colon tight junction proteins in T2DM mice. **(A)** Effect of MEP on T2DM induced endotoxemia. Serum LPS Level was measured by serum endotoxin (EU mL) Limulus amoebocyte lysate assay. **(B)** Effect of MEP on colon tight junction proteins (ZO-1, occludin, and slaudin-1) in T2DM mice. **(C–E)** Bar graph showing quantification of the western blot of the respective protein against β-actin as an internal control by Image J software. The data was obtained by three independent experiments and is expressed as mean SEM (*n* = 3). ^#^ indicates a significant difference compared with control (CON) group. ^###^*p* < 0.001 vs. control. * indicates a significant difference compared with the DM group. *p < 0.05, **p < 0.01 and ***p < 0.001 vs. DM. Data were presented as mean ± SEM.

In previous studies, HFD mice showed higher levels of pro-inflammatory dysbiosis of gut microbiota, leading to impaired intestinal permeability, elevated serum LPS, and metabolic endotoxemia, which eventually resulted in the expression of colon tight junction proteins (ZO-1, occludin, and claudin-1). Therefore, we evaluated the effect of MEP on serum LPS levels and colon tight junction protein expression in T2DM mice. In the DM group, we observed increased LPS levels, which had decreased significantly in the MEP-treated and control groups (Figure 8A). On the other hand, the colon tight junction proteins (ZO-1, occludin, and claudin-1) showed lower expression in the DM group but significantly higher expression in the MEP-treated and control groups (Figures 8B,C–E). These results demonstrated that MEP restores intestinal permeability and reduces endotoxemia in T2DM mice.

### *Morchella esculenta* polysaccharide improved colonic histological changes and modulated the expression of mucin-2 protein

**FIGURE 9:**
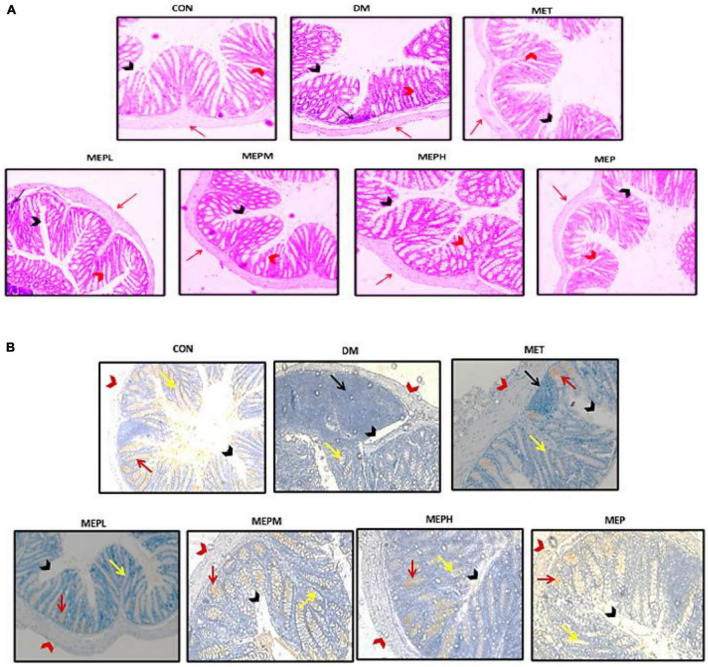
Effect of MEP on colonic histological changes by H&E (haematoxylin and eosin) staining and (MUC2) expression by IHC (immunohistochemistry) staining in T2DM mice. **(A)** Photomicrographs of distal colonic tissue, the (black arrows) indicate inflammatory cells, (black arrowhead) indicate mucosal space, (red arrow) epithelium surface and (red arrowhead) epithelial and goblet cells at magnification 20×. **(B)** Photomicrographs of the immunohistochemistry (red arrow) indicate MUC2 expression, (black arrows) indicated inflammatory cells, (black arrowhead) indicates mucosal space, (red arrows head) epithelium surface, and (yellow arrow) shows epithelial and goblet cells by the magnification of 20×.

Distal colon tissue histopathological assessment by H and E staining is shown in Figure 9A. The histological observation of colonic tissue in the DM group showed substantial loss and atrophy of crypts; irregular, short villi; a damaged epithelial barrier; and inflammatory cell infiltration. In contrast to the MEP- and MET-treated groups, these changes were reversed: the villi became well-demarcated regular and elongated with well-defined crypts and goblet cells, and the infiltration of inflammatory cells was alleviated. Moreover, to assess the mucus layer thickness of the epithelial gut barrier, we expressed mucin2 (MUC2) by IHC (Figure 9B). Notably, the expression of MUC2 decreased in the DM group compared to the others. These findings demonstrated that MEP may have the potential to ameliorate histological changes and alleviate MUC2 expression in colonic tissue.

## Discussion

Several clinical implications of mushroom polysaccharide have been investigated (23), including polysaccharide from *Morchella esculenta* (MEP) mushroom (13). However, the beneficial effect of MEP in particular on gut microbiota has not been studied. We demonstrated that MEP could affect hyperglycemia, insulin sensitivity, dyslipidemia, immunomodulation, and modulate gut microbiota.

We established a T2DM mouse model induced by HFD and STZ, based on a previously published strategy (24). The importance of the HFD- and STZ-induced T2DM model for drug effects has been studied thoroughly (25, 26). This method was widely used by researchers to create animal diabetic models for experimental purposes with clinical features similar to human diabetes (20, 27). Initially, T2DM mice show gradual emaciation, increased food and water intake, and decreased body weight (28). T2DM has been characterized by hyperglycemia, hyperinsulinemia, insulin resistance (HOMA-IR), and impaired glucose tolerance (29). In contrast to these studies, our findings demonstrated that MEP treatment in T2DM mice reduced the FBG level and improved glucose tolerance because OGTT is the most sensitive and common test for measuring the abnormality in glucose homeostasis (30). Previously, polysaccharides were revealed to reduce the rate of glucose diffusion because of their viscosity, which may absorb glucose (31). It is known that T2DM is associated with insulin resistance and increased serum insulin levels, which are required to control the glucose burden (28), and insulin resistance is responsible for the pathogenicity of T2DM and beta-cell failure (32). We observed that MEP administration could improve hyperinsulinemia and HOMA-IR, which is consistent with previous research findings.

The serum concentration of IL-6, IL-1β, and TNF-α were measured after 4 weeks of treatment with MEP in different groups to determine the immunoregulatory effect of MEP. Our results demonstrated that MEP administration regulated the abnormal secretion of pro-inflammatory cytokines, mainly IL-1β, IL-6, and TNF-α. Previously, the overproduction of IL-6 and TNF-α in T2DM patients led to the development of vascular inflammation and insulin resistance (33, 34). TNF-α is mostly expressed in adipose cells and is associated with insulin resistance (34), while highly expressed IL-6 causes cytotoxicity in pancreatic β cells (35). These findings indicated that MEP could regulate hyperglycemia and dyslipidemia in HFD- and STZ-induced T2DM mice by lowering the insulin resistance-related pro-inflammatory cytokines. Nevertheless, their molecular mechanisms remained unknown and needed further mechanistic studies to find out how MEP regulates glucose and lipid metabolism.

Moreover, gut microbiota dysbiosis is responsible for ulcerative colitis, inflammatory bowel diseases (IBD), and obesity (36), and is also associated with low-grade inflammation in T2DM (37). The dysbiosis of certain bacterial species that cause insulin resistance and metabolic syndromes have been reported (38, 39). In our findings, we found perturbations of bacterial abundance and richness in the DM group compared to the control group. However, when treated with MEP, bacterial abundance and diversity were augmented. The most abundant phyla of the gut microbial community were *Firmicutes, Actinobacteria, Proteobacteria*, and *Bacteroides.* Previously, many studies revealed that *Actinobacteria* increased (40, 41) while *Firmicutes* decreased in T2DM (42). Consistent with this result, our findings demonstrated an increased proportion of *Actinobacteria* and a decreased proportion of *Firmicutes* in the DM group. However, treatment with MEP potentially restored dysbiosis at the phylum level and showed similarity with the control and all MEP- and MET-treated groups. We further demonstrated the microbial community at the genus level. The probiotic *Lactobacillus* and *Prevotella* were found to be higher in the control, MEP-, and MET-treated groups. The growth of *Lactobacillus* and *Prevotella* was positively regulated by the MEP. These findings are consistent with previous studies because metformin increases the growth of *Lactobacillus* (43). Its enhancement has a hypolipidemic effect (44), whereas an increase in *Prevotella* abundance is associated with SCFA metabolites, which improve glucose metabolism (45).

The gut microbiota has a key role in digestion, which affects the state of diabetes, as is well documented. *Bacteroides vulgatus* and *Lactobacillus fermentum* were previously identified as cholesterol-lowering bacteria (44, 46). *Prevotella* is associated with improvement in glucose metabolism and the production of essential metabolites (45). *Prevotella* was used as a probiotic to reduce inflammation in adipose tissues (47). Increased *Actinobacteria* levels have been linked to elevated LPS levels, metabolic syndrome, insulin resistance, and obesity (40). *Clostridium bolteae* was found in high abundance as an opportunistic pathogen in diabetes, and *Clostridium* spp. are commensal bacteria in the colon that are harmful in large quantities (48). *Bifidobacterium* spp. (probiotic) has previously been shown to lower obesity (49).

The function of the organs, including the colon, were affected by hyperglycemia due to damaged blood vessels (50), so we examined the histopathological changes in colonic tissue in T2DM mice. Interestingly, our findings demonstrated that those treated with MEP showed amelioration in the histological alteration of colonic tissue. Previously, it was reported that T2DM and dysbiosis of gut microbiota led to impaired intestinal permeability (51), increased LPS levels and caused endotoxemia and metabolic disorders (52), which eventually affected the expression of colon tight junction proteins (ZO-1, occludin, and claudin-1) (51). Gut microbiota dysbiosis also led to decreased expression of the MUC2 protein, an important component of the gut barrier epithelium (53). Consistent with these findings, our results demonstrated that MEP treatment restored intestinal permeability by enhancing tight junctions (ZO-1, occludin, and claudin-1), regulating MUC2 protein expression, and reducing endotoxemia through lowering LPS levels in the T2DM mice.

Undoubtedly, diabetes mellitus is a systemic metabolic disorder that affects various tissues and organs in the body (54). The alteration of glucose metabolism in diabetic mice causes severe alterations in amino acid, lipid, and energy metabolism. This metabolic instability has been linked to a higher risk of vascular complications (55). Therefore, as diabetes progresses, it becomes increasingly important to identify metabolic changes all over the body (56). Moreover, the diverse functions played by altered microbial flora indicate that their dysbiosis would negatively impact metabolic pathways. Gut microbial activity have a direct or indirect impact on the host’s gastrointestinal function, as well as some influence on the auto-immune and metabolic systems.

Additionally, considering the significance of MEP in alleviating T2DM symptoms and modulating gut microbiota, we investigated these altered microbial flora to determine their unfavorable effects on functional metabolic pathways since T2DM is associated with altered metabolic pathways and metabolites (57). Amino acid and lipid metabolism, biosynthesis of other secondary metabolites, the immune and endocrine system, and signaling pathways were downregulated, while cardiovascular diseases, transport and catabolism, cell growth and death, and the digestive system were upregulated by MEP treatment. Recent findings connect dysregulation of bile acid biosynthesis to insulin resistance, dyslipidemia, and diabetes (58). However, natural indole alkaloids are on target for their anti-diabetic potential (59). In our study, the gene expression of indole biosynthesis and secondary bile acid biosynthesis was modulated by MEP treatment. These results demonstrated an altered functional metagenome among DM, control and MEP-treated groups.

## Conclusion

Altogether, polysaccharides extracted from MEP have the potential to reduce hyperglycemia and hyperlipidemia in T2DM mice. Treatment with MEP slowed the loss of body weight and improved polyphagia and polydipsia, glucose tolerance, and insulin resistance in T2DM mice. T2DM-induced inflammation was reduced by decreasing the production of pro-inflammatory cytokines. More importantly, MEP modulated the gut microbiota composition by reducing harmful bacterial taxa (*Actinobacteria, Corynebacterium, Facklamia*, and *Bacteroides*) while enhancing beneficial bacterial taxa (*Firmicutes*, and *Lactobacillus*). In addition, MEP has the potential to reduce endotoxemia, improve intestinal permeability, and ameliorate the metagenome of microbial communities by altering the functional metabolic pathways. We expect that the use of such bioactive polysaccharides for their prebiotic effects could be made feasible in the immediate future.

## Data availability statement

The original contributions presented in this study are included in the article/Supplementary material, further inquiries can be directed to the corresponding author/s.

## Ethics statement

The animal study was reviewed and approved by the Dalian Medical University Committee for animal experiments.

## Author contributions

YX and AR: conceptualization. AR, NS, and NF: methodology. AR, GA, and AK: software. AR and AK: formal analysis. AR, WZ, HS, WX, and SL: investigation. YX: resources, supervision and project administration. AR: manuscript writing. AR, GA, AG, BA, MA, and LW: review and editing. YX and LW: validation, visualization, and funding acquisition. All authors contributed to the article and approved the submitted version.
