# Supplementary material for: Morchella esculenta mushroom polysaccharide attenuates diabetes and modulates intestinal permeability and gut microbiota in a type 2 diabetic mice model
Source: Front Nutr. 2022 Oct 6;9:984695. doi: 10.3389/fnut.2022.984695 (PMC9582931; doi:10.3389/fnut.2022.984695)
Supplement: Supplementary file 1 [file Data_Sheet_1.PDF]

Supplementary Figures Legends

Fig S1: Box plot demonstrating the alpha diversity of gut microbiota in all groups, showing differences in microbial community richness and diversity. **(A)** Shannon **(B)**, Simpson and **(C)** Chao.

Fig S2: Heat map of microbiome composition with clustering analysis. Data of every taxonomic level were clustered following the degree of similarity among the group; the relative abundance of the taxonomic unit and the finding of clustering analysis were arranged individually according to taxonomic units.

Table S1: Summary of bacterial alpha diversity parameters of the different groups, including Shannon, Simpson, chao1, and ace.

Table S2: Summary of sequencing OUTs data analysis by 16sRNA gene sequencing in different treatment groups.

Table S3: Bacterial phylum percentage showing phyla present in the different treated groups.

Table S4: Bacterial genus percentage showing the bacterial community present at genus level in different groups of the study.

Table S5: Bacterial family percentage showing microbial community at family level in the different groups.

Supplementary Figures

Fig S1

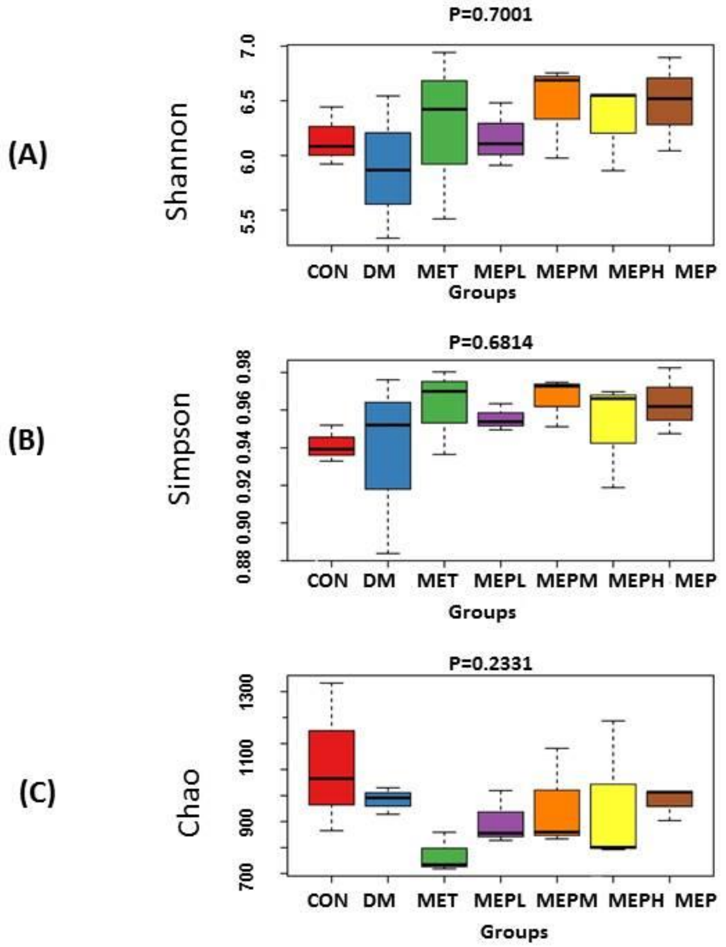

Fig S2

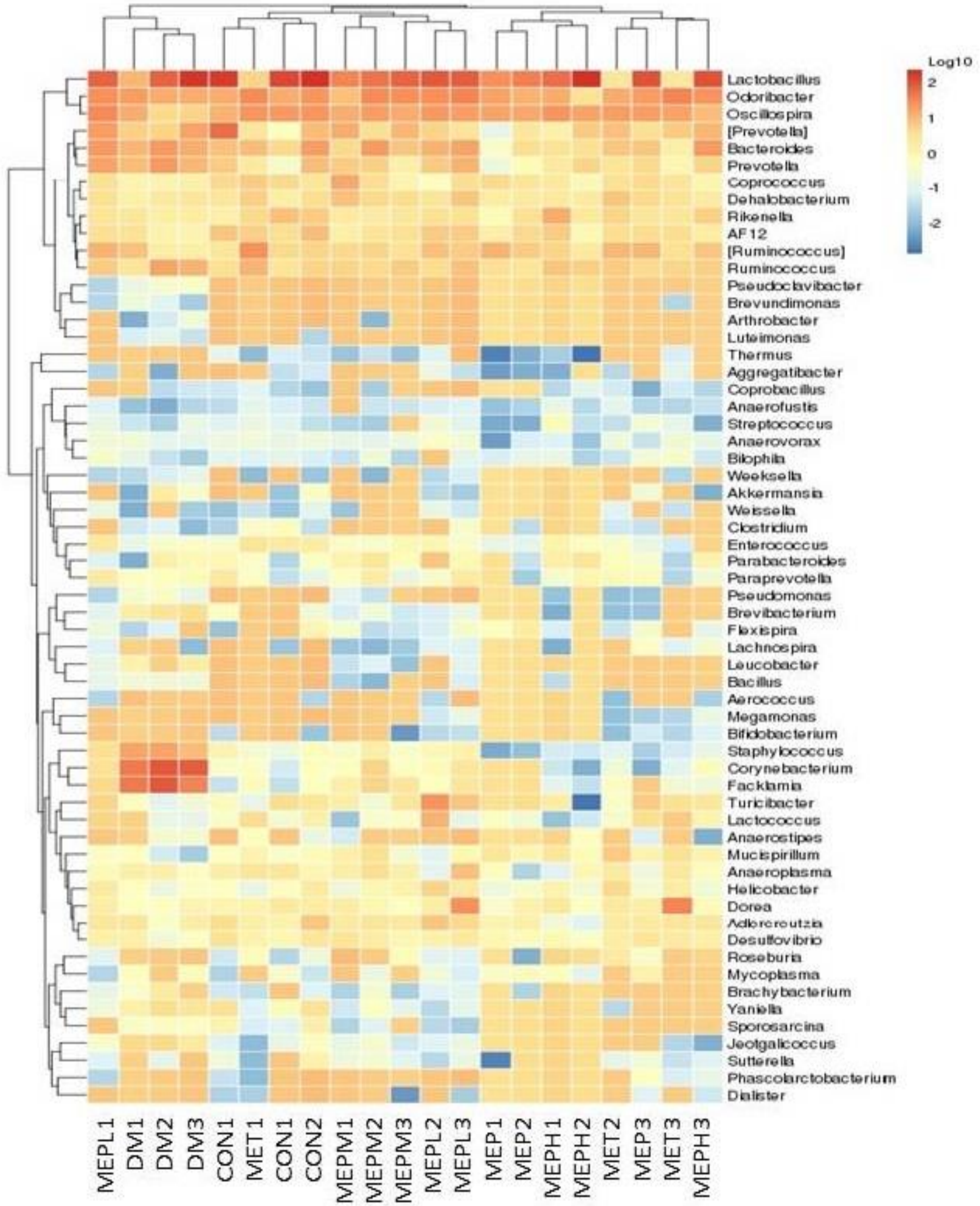
Log10
2
1
0
-1
-2
Lactobacillus
Odoribacter
Oscillospira
[Prevotella]
Bacteroides
Prevotella
Coprococcus
Dehalobacterium
Rikenella
AF12
[Ruminococcus]
Ruminococcus
Pseudoclavibacter
Brevundimonas
Arthrobacter
Luteimonas
Thermus
Aggregatibacter
Coprobacillus
Anaerofustis
Streptococcus
Anaerovorax
Bilophila
Weeksella
Akkermansia
Weissella
Clostridium
Enterococcus
Parabacteroides
Paraprevotella
Pseudomonas
Brevibacterium
Flexispira
Lachnospira
Leucobacter
Bacillus
Aerococcus
Megamonas
Bifidobacterium
Staphylococcus
Corynebacterium
Facklamia
Turicibacter
Lactococcus
Anaerostipes
Mucispirillum
Anaeroplasma
Helicobacter
Dorea
Adlercreutzia
Desulfovibrio
Roseburia
Mycoplasma
Brachybacterium
Yaniella
Sporosarcina
Jeotgalicoccus
Sutterella
Phascolarctobacterium
Dialister
MEPL1
DM1
DM2
DM3
CON1
MET1
CON1
CON2
MEPM1
MEPM2
MEPM3
MEPL2
MEPL3
MEP1
MEP2
MEPH1
MEPH2
MET2
MEP3
MET3
MEPH3

Supplementary Tables

**Table S1: Summary of bacterial alpha diversity**					
**Groups**	**Shannon**	**SImpson**	**Chao1**	**Ace**	**Goods_coverage**
**CON1**	6.082	0.932	1432.62	1408.24	0.998
**CON2**	6.444	0.951	1065.46	1059.08	0.998
**CON3**	6.775	0.975	1231.73	1233.77	0.998
**DM1**	6.544	0.976	1029.68	977.68	0.998
**DM2**	5.865	0.952	991.51	1001.11	0.998
**DM3**	5.244	0.883	928.26	910.5	0.998
**MET1**	6.941	0.98	859.52	813.95	0.999
**MET2**	6.421	0.969	734.46	726.74	0.999
**MET3**	6.209	0.919	969.81	952.97	0.998
**MEPL1**	5.909	0.949	827.48	836.03	0.998
**MEPL2**	6.478	0.963	1018.71	963.95	0.998
**MEPL3**	6.105	0.953	855.83	849.16	0.999
**MEPM1**	6.687	0.974	859.7	873.99	0.999
**MEPM2**	5.976	0.951	833.32	819.88	0.999
**MEPM3**	6.753	0.972	1181.83	1162.95	0.998
**MEPH1**	6.547	0.969	792.5	775.33	0.999
**MEPH2**	6.011	0.963	763.31	761.94	0.999
**MEPH3**	6.55	0.966	1256.54	1276.95	0.998
**MEP1**	6.518	0.961	1013.37	1009.74	0.998
**MEP3**	6.895	0.982	1014.49	1015.7	0.998
**MEP4**	6.042	0.947	904.5	915.14	0.998

Table S2:

Summary of Sequencing OUTs data

Groups	Sample Size	Singleton%	Chimeras%	Clean_tags	OUT,S	Coverages
**CON1**	138897	15.94	2.57	128654	1289	1
**CON2**	130657	9.22	0.54	127999	931	1
**CON3**	124412	10.9	0.67	120672	1029	1
**DM1**	133594	9.13	11.69	115378	799	1
**DM2**	138997	9.4	10.35	121045	842	1
**DM3**	137241	8.57	4.14	129341	781	1
**MET1**	137711	10.78	0.21	135521	732	1
**MET2**	139648	7.88	0.35	137761	677	1
**MET3**	135937	9.58	3.85	128271	805	1
**MEPL1**	133483	8.56	0.34	13143	592	1
**MEPL2**	128339	9.19	0.31	126099	715	1
**MEPL3**	137891	9.25	0.73	135165	744	1
**MEPM1**	133790	9.44	0.54	131339	779	1
**MEPM2**	139191	9.22	0.99	135867	718	1
**MEPM3**	132097	9.68	2.75	125969	957	1
**MEPH1**	105151	33.87	0.05	101971	712	1
**MEPH2**	136301	12.17	0.51	132190	1078	1
**MEPH3**	103997	30.6	0.31	100760	699	1
**MEP1**	129776	9.2	0.39	127191	881	1
**MEP2**	138255	10.35	0.35	135218	885	1
**MEP3**	134550	9.38	0.41	131827	775	1

Table S3: **Bacterial phylum percentage**

Groups	Firmicutes	Bacteroidetes	Proteobacteria	Actinobacteria	Tenerictus	TM7	Deferribacteres	Cyanobacteria	Verrucomicrobi
**CON**	64.07	31.05	1.66	0.67	0.65	1.58	0.08	0.16	0
**DM**	49.39	33.17	1.6	16.46	0.84	0.14	0.08	0.13	0.14
**MET**	67.35	24.54	5.6	0.28	0.74	1.04	0.38	0.05	0
**MEPL**	67.51	28.23	1.76	0.85	0.77	0.62	0.2	0.03	0.01
**MEPM**	62.48	33.16	1.92	0.74	0.43	0.92	0.23	0.04	0
**MEPH**	62.17	29.4	3.08	0.21	0.64	0.92	0.3	0.01	0
**MEP**	70.44	25.16	2.68	0.31	0.33	0.6	0.38	0.02	0.034

Table S4: Bacterial genus percentage

Groups	Lac_Lactobacillus	Odoribacter	Oscillospira	Corynebacterium	Bacteroides
**CON**	69.12	5.7	7.01	0.09	1.88
**DM**	32.09	4.92	3.57	25.2	4.82
**MET**	47.41	12.64	15.6	1.53	3.65
**MEPL**	67.64	8.21	9.3	0.32	4.13
**MEPM**	64.13	11.95	8.09	0.74	3.36
**MEPH**	59.12	12.63	9.97	4.38	1.44
**MEP**	61.62	12.24	12.39	0	2.16
**Groups**	**Par_Prevotella**	**Facklamia**	**Lac_Ruminococcus**	**Rum_Ruminococcus**	**Pre_Prevotella**
**CON**	11.05	0.11	0.96	1.22	2.78
**DM**	1.62	18.98	0.87	3.7	4.15
**MET**	3.72	0.2	11.32	3.11	0.77
**MEPL**	3.39	0.31	2.69	1.7	2.26
**MEPM**	5.32	0.83	2.11	1.85	1.58
**MEPH**	3.2	0.05	3.27	3.42	0
**MEP**	1.24	0	7.09	2.54	0.86

Table S5:

	Bacterial family percentage					
**Group**	**Lactobacillaceae**	**S24-7**	**Lachnospiraceae**	**Ruminococcaceae**	**Rikenellaceae**	**Odoribacteraceae**
**CON**	39.92	25.9	8.63	6.1	8.4	3.3
**DM**	10.93	26.02	7.45	9.5	4.3	5.5
**MET**	43.96	12.5	6.48	10.85	8.7	8.8
**MEPL**	50.42	16.43	9.09	8.47	4.65	5.6
**MEPM**	32.82	29.34	11.63	9	5.06	6.11
**MEPH**	47.42	22.5	6.29	11.1	4.79	1.27
**MEP**	34.77	9.34	17.04	12.88	16.94	6.07
**Group**	**Corynebacteriaceae**	**Aerococcaceae**	**Helicobacteraceae**	**Paraprevotellaceae**	**Enterococcaceae**	
**CON**	0.05	0.22	0.75	6.5	0.1	
**DM**	17.4	15.09	1.06	2.49	0.07	
**MET**	3.33	0.39	0.05	3.78	1.03	
**MEPL**	0.24	0.36	1.5	2.6	0.49	
**MEPM**	0.73	0.5	1.7	2.8	0.16	
**MEPH**	0.32	0.02	3.24	2.8	0.23	
**MEP**	0	0	2.2	0.63	0.04	
